# Dellaglioa kimchii sp. nov., a novel lactic acid bacterium isolated from kimchi

**DOI:** 10.1099/ijsem.0.006829

**Published:** 2025-06-27

**Authors:** Hye In Ko, So-Rim Kim, Chae-Rim Jeong, Misun Yun, Sohee Nam, Jong-Bang Eun, Tae-Woon Kim

**Affiliations:** 1 Technology Innovation Research Division, World Institute of Kimchi, Gwangju 61755, Republic of Korea; 2 Department of Integrative Food, Bioscience and Biotechnology, Chonnam National University, Gwangju 61186, Republic of Korea; 3 Department of Integrative Biological Sciences, Chosun University, Gwangju 61452, Republic of Korea

**Keywords:** *Dellaglioa kimchii*, kimchi, lactic acid bacteria, new taxon, P0083, taxonomy

## Abstract

A short, rod-shaped, Gram-positive, catalase-negative and facultative anaerobic bacterium, designated strain P0083^T^, was isolated from kimchi, a traditional Korean fermented vegetable. Phylogenetic analysis based on the nearly complete 16S rRNA and *recA* gene sequences placed the isolate within a distinct group from other closely related species within the *Dellaglioa* genus. Under anaerobic conditions on de Man, Rogosa and Sharpe agar, the bacterium formed beige, circular colonies of moderate size. Growth occurred between 4 and 25 °C, with optimal growth at 20 °C and limited growth at 30 °C. The bacterium grew at pH 5–8 and tolerated 3% NaCl. The main cellular fatty acids included C16 : 0 fatty acid methyl esters (FAME) and C18 : 1 CIS 9 FAME. Digital DNA–DNA hybridization confirmed that strain P0083^T^ represents a novel genomic species, with *Dellaglioa* species sharing less than 84.22% DNA homology with strain P0083^T^. The genomic, phenotypic and biochemical characteristics of *Dellaglioa kimchii* support the hypothesis that strain P0083^T^ (=JCM 37383^T^=KCTC 25916^T^) represents a novel species, *Dellaglioa kimchii* sp. nov., with P0083^T^ as the type strain.

## Introduction

Kimchi is the representative Korean fermented vegetable food that is well known worldwide. It is made from salted vegetables combined with various other ingredients, including ginger, green onion, red pepper powder, garlic and seafood [1,2]. The pH of kimchi typically decreases from ~5.8 to 4.0 during fermentation as lactic acid bacteria (LAB) produce organic acids [3]. Various LAB play an important role during kimchi fermentation, influencing flavour and demonstrating health-promoting characteristics [4,5]. Although kimchi fermentation typically occurs below 10 °C, isolation of lactic acid bacteria has been commonly performed at temperatures between 30 and 37 °C for 24–72 h, which limits the isolation of psychrotrophic LAB such as *Leuconostoc gelidum*, *Leuconostoc gasicomitarum* and *Leuconostoc inhae* because they can barely grow at 30 °C [6,8] and are commonly isolated from kimchi when lower temperatures (10 and 20 °C) are applied [1]. Strain P0083^T^ was isolated during an investigation of psychrotrophic LAB associated with kimchi fermented below 10 °C. This strain was initially clustered in *Dellaglioa algida* by 16S rRNA gene sequence comparison. However, subsequent whole-genome sequence analysis indicated that it might represent a novel *Dellaglioa* species.

*Dellaglioa* is a genus of LAB that was distinguished from *Lactobacillus* through a taxonomic revision by Zheng *et al*. [9]. To date, two species within this genus have been described, namely *D. algida* and *Dellaglioa carnosa. Dellaglioa* spp. are adapted to cold environments, are associated with animals and are prevalent in various meat products [10,11]. However, there is little research on the presence of *D. algida* from plant-based food sources such as kimchi [1].

In this study, one LAB strain, designated P0083^T^, isolated from kimchi, was examined by a polyphasic taxonomic approach to determine its precise taxonomic position. This was confirmed by further phenotypic, genotypic and chemotaxonomic analyses. Thus, strain P0083^T^ is proposed as *Dellaglioa kimchii* sp. nov.

## Methods

Kimchi was made with kimchi cabbage produced in Cheongju, Chungcheongbuk-do (36° 44′ 15″ N 127° 30′ 44″ E), Republic of Korea. The pH of the kimchi was 4.01±0.01 when the strain was isolated. The kimchi was ground and filtered using sterile gauze. Afterwards, 25 ml of the kimchi extract was homogenized with 225 ml of sterile saline, and the filtrate was serially diluted 1:10. The diluted solution was spread onto de Man, Rogosa, and Sharpe (MRS) agar plates and incubated at 20 ℃ for 48 h anaerobically (Gaspack; BD, NJ, USA). The isolated colony was subcultured thrice to obtain the pure strain. Strain P0083^T^ was preserved in 10% skim milk stock at −80 ℃. Based on *recA* gene clustering in the phylogenetic tree, *D. algida* KACC 12389^T^ (=DSM 15638^T^), *D. carnosa* DSM 114968^T^, *Ligilactobacillus apodemi* KACC 12406^T^ (=DSM 16634^T^), *Ligilactobacillus murinus* KACC 12432^T^ (=DSM 20452^T^) and *Ligilactobacillus animalis* KACC 12441^T^ (=DSM 20602^T^) were selected as reference strains and obtained from the Korean Agricultural Culture Collection (KACC) and German Collection of Microorganisms and Cell Cultures GmbH.

Cell morphology was observed using cells cultivated on MRS agar at 20 ℃ for 48 h. Gram staining was conducted with a Gram Color Kit (MBcell, Seoul, Republic of Korea), and the catalase test was performed with a 3% hydrogen peroxide solution. The carbohydrate fermentation tests were evaluated using the API 50CH kit (bioMérieux, France), and enzyme activity was assessed with the API Zym kit (bioMérieux, France) following the manufacturer’s instructions. Lactic acid isomers were analysed using the d-/l-lactic acid assay kit (Megazyme, Bray, Ireland).

To assess the optimal growth temperature, cultures were incubated in MRS broth at 4, 10, 15, 20, 25 and 30 ℃ for 48 h, and optical density was measured at 600 nm with a spectrophotometer (Infinite M200PRO Nanoquant, Tecan, Switzerland). Salt tolerance and the growth pH range were evaluated in MRS agar with NaCl concentrations of 3, 4 and 6.5% and at pH 3, 4, 5, 6, 7 and 8 for 72 h. The fatty acid profiles were analysed using the Sherlock Microbial Identification System (Agilent 8890 GC system; software: MIDI 6.5 version) provided by the microbial identification service at the Korean Collection for Type Cultures (KCTC).

**Fig. 1.**
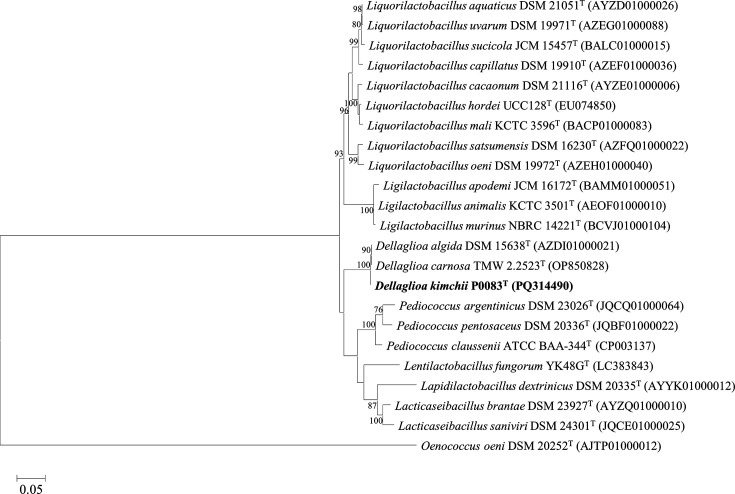
Phylogenetic tree of *D. kimchii* P0083^T^ and closely related species using the ML algorithm based on an alignment of 1,483 bp nucleotides of 16S rRNA gene sequences. Bootstrap values, calculated from 1,000 replicates, are displayed on the nodes only when 70% or higher. *Oenococcus oeni* DSM 20252^T^ was used as the outgroup.

The 16S rRNA of the strain was amplified by Macrogen (Seoul, Republic of Korea) using primers 27F (5′-AGAGTTTGATCMTGGCTCAG-3) and 1492R (5′-TACGGYTACCTTGTTACGACTT-3′). Subsequently, it was sequenced using primers 785F (5′-GGATTAGATACCCTGGTA-3′) and 907R (5′-CCGTCAATTCMTTTRAGTTT-3′). The similarity of the obtained sequence was analysed using EzBioCloud (https://www.ezbiocloud.net). Sequences were also compared with the 16S rRNA sequences of closely related strains obtained from EzBioCloud. Phylogenetic analysis was performed using the mega11 program [12]. All sequences were aligned using the clustalw [13] algorithm, and a phylogenetic tree was constructed using the maximum likelihood (ML) estimation method [14] and the neighbour-joining (NJ) [15] and maximum parsimony (MP) [16] algorithms with 1,000 bootstrap replicates (Figs 1 and S1, available in the online Supplementary Material). The *recA* gene sequence of strain P0083^T^ was obtained from the draft genome and used to construct phylogenetic trees. The *recA* gene sequences from closely related strains were obtained from the National Center for Biotechnology Information (NCBI). The phylogenetic tree for the *recA* gene sequences was constructed similarly to that of the 16S rRNA gene phylogenetic analysis.

Whole-genome sequencing of strain P0083^T^ was performed by CJ Bioscience (Seoul, Republic of Korea). Genomic DNA extraction was performed using the QIAGEN MagAttract HMW DNA kit (QIAGEN, Hilden, Germany) according to the manufacturer’s instructions. The extracted genome DNA was sequenced using the PacBio and Illumina MiSeq platforms. The genome of strain P0083^T^ was constructed *de novo* using PacBio and MiSeq sequencing data. Hybrid assembly was performed using Unicycler version 0.4.3. The EzBioCloud genome database was utilized for gene-finding and as the functional annotation pipeline of whole-genome assemblies. Prodigal 2.6.2 was used to predict protein-coding sequences (CDSs) [17]. tRNAscan-SE 1.3.1 was employed to search genes coding tRNA [18]. To search rRNA and other non-coding RNAs, a covariance model search was conducted using the Rfam 12.0 database [19]. Clustered regularly interspaced short palindromic repeats were detected with PilerCR 1.06 [20] and CRT 1.2 [21]. For additional functional annotation, the predicted CDSs were analysed against the SwissProt [22], Kyoto Encyclopedia of Genes and Genomes [23] and SEED [24] databases using ublast [25].

For comparative genomic analysis, the genome sequences and annotation files of type strains closely related to strain P0083^T^, including *D. algida* KACC 12389^T^ (=DSM 15638^T^), *D. carnosa* DSM 114968^T^ (=TMW 2.2523^T^), *Ligilactobacillus apodemi* KACC 12406^T^ (=DSM 16634^T^), *Ligilactobacillus murinus* KACC 12432^T^ (=DSM 20452^T^) and *Ligilactobacillus animalis* KACC 12441^T^ (=DSM 20602^T^), were obtained from NCBI GenBank.

Comparative genomic analysis was performed using average nucleotide identity (ANI) and digital DNA–DNA hybridization (dDDH). dDDH was calculated with the Genome-to-Genome Distance Calculator (https://ggdc.dsmz.de/ggdc.php) [26], and ANI was analysed using the ANI Calculator from EZBioCloud [27]. Whole-genome-based taxonomic analysis was performed using the Type (Strain) Genome Server – a free bioinformatics platform (https://tygs.dsmz.de) based on the Genome BLAST Distance Phylogeny (GBDP).

## Phenotypic, biochemical and chemotaxonomic analysis

According to the results of the API 50CH test, strain P0083^T^ did not ferment d-galactose or d-fructose, whereas *D. algida* and *D. carnosa* did (Table 1). Regarding enzyme production, strain P0083^T^ showed no acid phosphatase activity, which differed from *D. algida* and *D. carnosa* (Table 2). Fatty acid profile analysis identified C16 : 0 fatty acid methyl esters (FAME) and C18 : 1 CIS 9 FAME to be the most abundant fatty acids, consistent with other *Dellaglioa* species (Table 3). Additionally, strain P0083^T^ exhibited optimal growth at 20 °C, a characteristic similar to that observed in other *Dellaglioa* species. Strain P0083^T^ failed to grow at a NaCl concentration of ≥4% but grew at pH 5–8. While the same characteristics were observed for *D. carnosa*, *D. algida* exhibited similar behaviour, except for slight growth at 4% NaCl.

**Table 1.** Carbohydrate fermentation of strain P0083^T^ and related type strains

Fermentation (API 50CH)	1	2	3	4	5	6
Control	−	−	−	−	−	−
Glycerol	−	−	−	−	−	−
Erythritol	−	−	−	−	−	−
d-Arabinose	−	−	−	−	−	−
l-Arabinose	+	+	−	−	+	−
d-Ribose	−	+	−	+	w	−
d-Xylose	+	−	w	−	−	−
l-Xylose	−	−	−	−	−	−
d-Adonitol	−	−	−	−	−	−
Methyl-*β*-d-xylopyranoside	−	−	−	−	−	−
d-Galactose	−	+	w	+	+	+
d-Glucose	+	+	+	+	+	+
d-Fructose	−	+	w	+	+	+
d-Mannose	w	+	+	+	+	+
l-Sorbose	−	−	−	−	−	−
Rhamnose	−	−	−	−	−	−
Dulcitol	−	−	−	−	−	−
Inositol	−	−	−	−	−	−
d-Mannitol	−	−	−	−	+	−
d-Sorbitol	−	−	−	−	−	−
Methyl *α*-d-mannopyranoside	−	−	−	−	−	−
Methyl *α*-d-glucopyranoside	−	−	−	−	−	−
*N*-Acetylglucosamine	w	+	w	+	+	+
Amygdalin	−	−	−	−	−	w
Arbutin	−	+	−	+	+	+
Esculin	+	+	+	+	+	+
Salicin	−	+	−	+	+	+
d-Cellobiose	−	w	−	+	+	+
d-Maltose	−	−	+	+	+	+
d-Lactose	−	−	−	+	+	+
d-Melibiose	−	−	−	+	+	+
Sucrose	−	−	−	+	+	+
d-Trehalose	−	w	−	+	−	−
Inulin	−	−	−	−	−	−
d-Melezitose	−	−	−	−	−	−
d-Raffinose	−	−	−	+	+	+
Amidon (starch)	−	−	−	−	−	−
Glycogen	−	−	−	−	−	−
Xylitol	−	−	−	−	−	−
Gentiobiose	−	−	−	−	+	+
d-Turanose	−	−	−	−	w	−
d-Lyxose	−	−	−	−	−	−
d-Tagatose	−	−	−	−	−	−
d-Fucose	−	−	−	−	−	−
l-Fucose	−	−	−	−	−	−
d-Arabitol	−	−	−	−	−	−
l-Arabitol	−	−	−	−	−	−
Gluconate	−	−	−	−	−	−
2-Keto-gluconate	−	−	w	−	−	−
5-Keto-gluconate	−	−	−	−	−	−

1, *D. kimchii* P0083T; 2, *D. algida* KACC 12389T; 3, *D. carnosa* DSM 114968T; 4, *Ligilactobacillus apodemi* KACC 12406T; 5, *Ligilactobacillus murinus* KACC 12432T; 6, *Ligilactobacillus animalis* KACC 12441T.

**Table 2.** Enzyme reactions of strain P0083^T^ and related type strains

Enzyme reaction (API Zym)	1	2	3	4	5	6
Alkaline phosphatase	−	−	−	−	−	−
Esterase (C4)	−	−	−	−	−	−
Esterase lipase (C8)	−	−	−	−	−	−
Lipase (C14)	−	−	−	−	−	−
Leucine arylamidase	+	+	+	+	+	−
Valine arylamidase	+	+	+	−	+	−
Crystine arylamidase	−	−	−	−	−	−
Trypsin	−	−	−	−	−	−
*α*-Chymotrypsin	−	−	−	−	−	−
Acid phosphatase	−	+	+	−	+	+
Naphthol-AS-BI-phosphohydrolase	w	+	w	−	+	−
*α*-Galactosidase	−	−	−	−	+	−
*β*-Galactosidase	+	+	+	−	+	−
*β*-Glucuronidase	−	−	−	−	−	−
*α*-Glucosidase	−	−	−	−	−	−
*β*-Glucosidase	−	−	−	−	−	−
*N*-Acetyl-*β*-glucosaminidase	−	−	−	−	−	−
*α*-Mannosidase	−	−	−	−	−	−
*α*-Fucosidase	−	−	−	−	−	−

1, *D. kimchii* P0083T; 2, *D. algida* KACC 12389T; 3, *D. carnosa* DSM 114968T; 4, *Ligilactobacillus apodemi* KACC 12406T; 5, *Ligilactobacillus murinus* KACC 12432T; 6, *Ligilactobacillus animalis* KACC 12441T.

**Table 3.** Differentiating characteristics between strain P0083^T^ and related type strains

Characteristic	1	2	3
**Fatty acid**			
C10 : 0 ISO FAME	0.2	nr	nr
C10 : 0 FAME	0.7	0.4	0.4
C12 : 0 FAME	nr	nr	0.5
C14 : 0 FAME	3.1	2.2	1.4
C15 : 0 FAME	0.3	nr	nr
C16 : 0 FAME	23.3	17.3	11.1
C16 : 1 CIS 9 DMA	nr	nr	0.4
18 : 1 CIS 9 FAME	68.8	72.7	73.1
C18 : 0 FAME	0.7	1.2	2.4
UN 18.199 18:Oa DMA	0.9	2.0	3.0
18 : 1 CIS 11 DMA	0.4	1.5	2.4
C20 : 0 FAME	nr	nr	1.5
Summed feature 10 (18 : 1c11/t9/t6 FAME, UN 17.834)	1.0	1.5	1.7
Summed feature 12 (UN 18.622, 19 : 0 ISO FAME)	0.5	1.3	2.3
Growth at (°C), recommended (parentheses)			
4,10, 15, 20, 25	+(20 °C)	+(20 °C)	+(20 °C)
30	−	−	−
NaCI (%)			
3	+	+	+
4	−	w	−
6.5	−	−	−
**pH**			
**3**	−	−	−
**4**	−	−	−
**5**	+	+	+
**6**	+	+	+
**7**	+	+	+
**8**	+	+	+

1, *D. kimchii* P0083T; 2, *D. algida* KACC 12389T; 3, *D. carnosa* DSM 114968T.

nr, not reported.

## Phylogenetic analysis

**Fig. 2.**
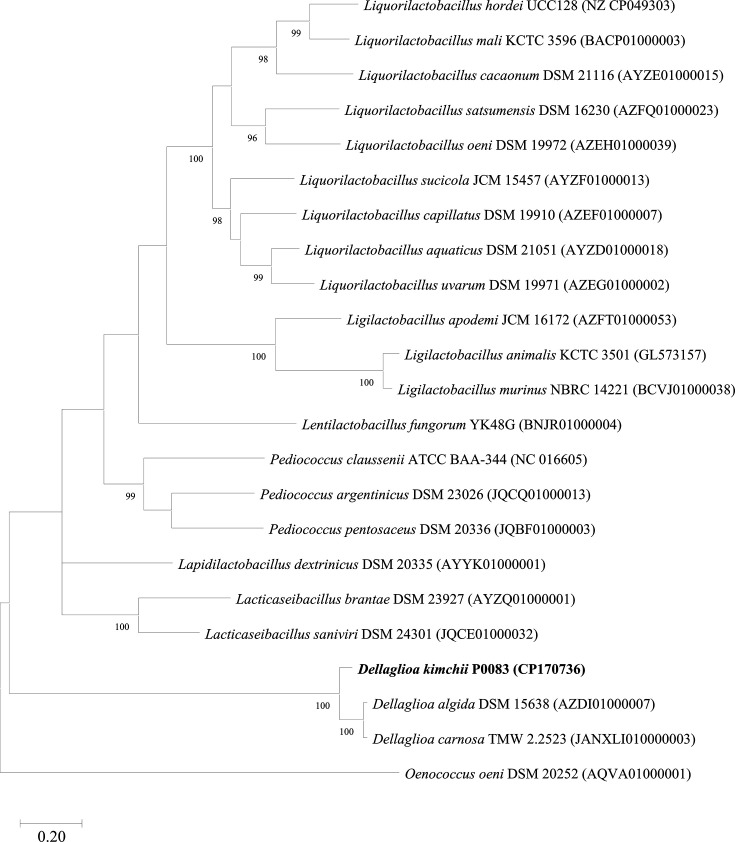
Phylogenetic tree of *D. kimchii* P0083^T^ and closely related species using the ML algorithm based on *recA* gene sequences. Bootstrap values, calculated from 1,000 replicates, are displayed on the nodes only when 70% or higher. *Oenococcus oeni* DSM 20252^T^ was used as the outgroup.

The 16S rRNA sequence of strain P0083^T^ demonstrated that it is most closely related to members of the genus *Dellaglioa* (Figs 1 and S1). Strain P0083^T^ exhibited 99.87% similarity with *D. algida* DSM 15638^T^ and *D. carnosa* DSM 114968^T^, but <95% similarity with other strains. In the ML, NJ and MP analyses, strain P0083^T^ clustered closest with *D. algida* DSM 15638^T^ and *D. carnosa* DSM 114968^T^, distinguishing it from other species. Phylogenetic analysis of the *recA* gene also indicated that strain P0083^T^ clustered closely with the genus *Dellaglioa*, further distinguishing it from other species (Figs 2 and S2).

## Genomic analysis

Strain P0083^T^ comprised two contigs with a depth coverage of 1,066.12 ×, a genome size of 1,570,579 bp, guanine plus cytosine (G+C) content of 34.2 mol% and N50 length of 1,516,742 bp. The genome comprised 1,488 protein-coding genes, 60 tRNA genes and 16 rRNA genes.

**Fig. 3.**
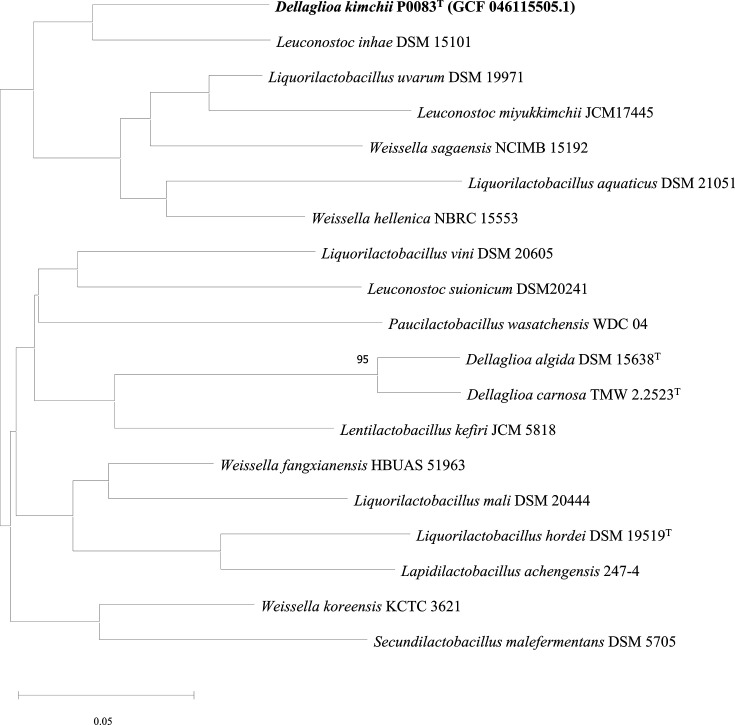
GBDP tree (whole-genome sequence-based) of *D. kimchii* P0083^T^ and closely related species.

Strain P0083^T^ shared the highest similarity with *D. carnosa* DSM 114968^T^, with a dDDH value range from 22.0% to 27.3% compared to other strains (Table 4), lower than the bacterial species differentiation threshold of 70% [28,29]. Additionally, the ANI value of strain P0083^T^ was also highest when compared with *D. carnosa* DSM 114968^T^, ranging from 69.02% to 84.22% in comparison with selected other strains, thereby satisfying the bacterial species differentiation threshold of 95%–96% [28,29]. Contrary to the 16S rRNA results, the phylogenetic tree analysis based on the whole genome identified *Leuconostoc inhae* DSM 15101^T^ as closest to strain P0083^T^, while *D. carnosa* DSM 114968^T^ and *D. algida* DSM 15638^T^ were distant (Fig. 3). These results confirm that strain P0083^T^ isolated from kimchi represents a new *Dellaglioa* species, designated *D. kimchii* sp. nov.

**Table 4.** The orthologous ANI and dDDH values of strain P0083^T^ and related type strains

Strain	P0083^T^	*D. algida*DSM 15638^T^	*D. carnosa*DSM 114968^T^	*Ligilactobacillus apodemi*DSM 16634^T^	*Ligilactobacillus animalis*DSM 20602^T^	*Ligilactobacillus murinus*DSM 20452^T^
P0083^T^	–	27.20	27.30	22.00	25.70	22.90
*D. algida* DSM 15638^T^	84.02	–	63.20	18.40	20.30	21.20
*D. carnosa* DSM 114968^T^	84.22	95.42	–	20.00	20.10	21.50
*Ligilactobacillus apodemi* DSM 16634^T^	69.05	69.07	69.27	–	20.70	21.50
*Ligilactobacillus animalis* DSM 20602^T^	69.02	68.81	68.91	76.80	–	45.60
*Ligilactobacillus murinus* DSM 20452^T^	68.76	68.56	68.76	77.14	91.44	–

The upper metric represents dDDH, while the lower metric represents ANI values.

In conclusion, strain P0083^T^, isolated from kimchi, is identified as a new species within the genus *Dellaglioa*, supported by phenotypic, biochemical, chemotaxonomic, phylogenetic and genomic analyses. The proposed name for this species is *D. kimchii* sp. nov., with P0083^T^ as the type strain.

## Description of *Dellaglioa kimchii* sp. nov.

*Dellaglioa kimchii* sp. nov. (kim’chi.i. N.L. gen. n. *kimchii*, from kimchi, a traditional Korean fermented vegetable food).

The cells are Gram-positive, facultatively anaerobic, catalase-negative and short rod-shaped. It grows on MRS agar under facultative anaerobic conditions, forming colonies of moderate size. The colonies are beige and circular. The temperature range for growth is 4–25 °C, with optimal growth at 20 °C. It does not grow at 30 °C. It can grow within the pH range of 5–8 and tolerates NaCl concentrations of up to 3%. In the API 50CH test, this strain is positive for l-arabinose, d-xylose, d-glucose and aesculin and exhibits weakly positive reactions for d-mannose and *N*-acetylglucosamine. In the API Zym test, it is positive for leucine arylamidase, valine arylamidase and *β*-galactosidase and is weakly positive for naphthol-AS-BI-phosphohydrolase. The main cellular fatty acids are C16 : 0 FAME and C18 : 1 CIS 9 FAME. The type strain is strain P0083^T^ (JCM 37383=KCTC 25916), isolated from kimchi obtained from a kimchi production facility in Cheongju, Chungcheongbuk-do, South Korea, and has a DNA G+C content of 34.2 mol%. The 16S rRNA gene sequence of the type strain is registered in the NCBI GenBank database under the accession number PQ314490, and the whole-genome sequences are available under CP170736 and CP170737.

## Supplementary material

10.1099/ijsem.0.006829 Uncited Supplementary Material 1.

## Abbreviations

ANI: average nucleotide identity
CDSs: coding sequences
dDDH: digital DNA–DNA hybridization
FAME: fatty acid methyl esters
GBDP: genome BLAST distance phylogeny
G+C: guanine plus cytosine
KACC: Korean Agricultural Culture Collection
KCTC: Korean Collection for Type Cultures
LAB: lactic acid bacteria
ML: maximum likelihood
MP: maximum parsimony
MRS: de Man, Rogosa and Sharpe
NCBI: National Center for Biotechnology Information
NJ: neighbor-joining
