# Supplementary material for: Dellaglioa kimchii sp. nov., a novel lactic acid bacterium isolated from kimchi
Source: Int J Syst Evol Microbiol. 2025 Jun 27;75(6):006829. doi: 10.1099/ijsem.0.006829 (PMC12281850; doi:10.1099/ijsem.0.006829)
Supplement: Uncited Supplementary Material 1. [file ijsem-75-06829-s001.pdf]

(A)

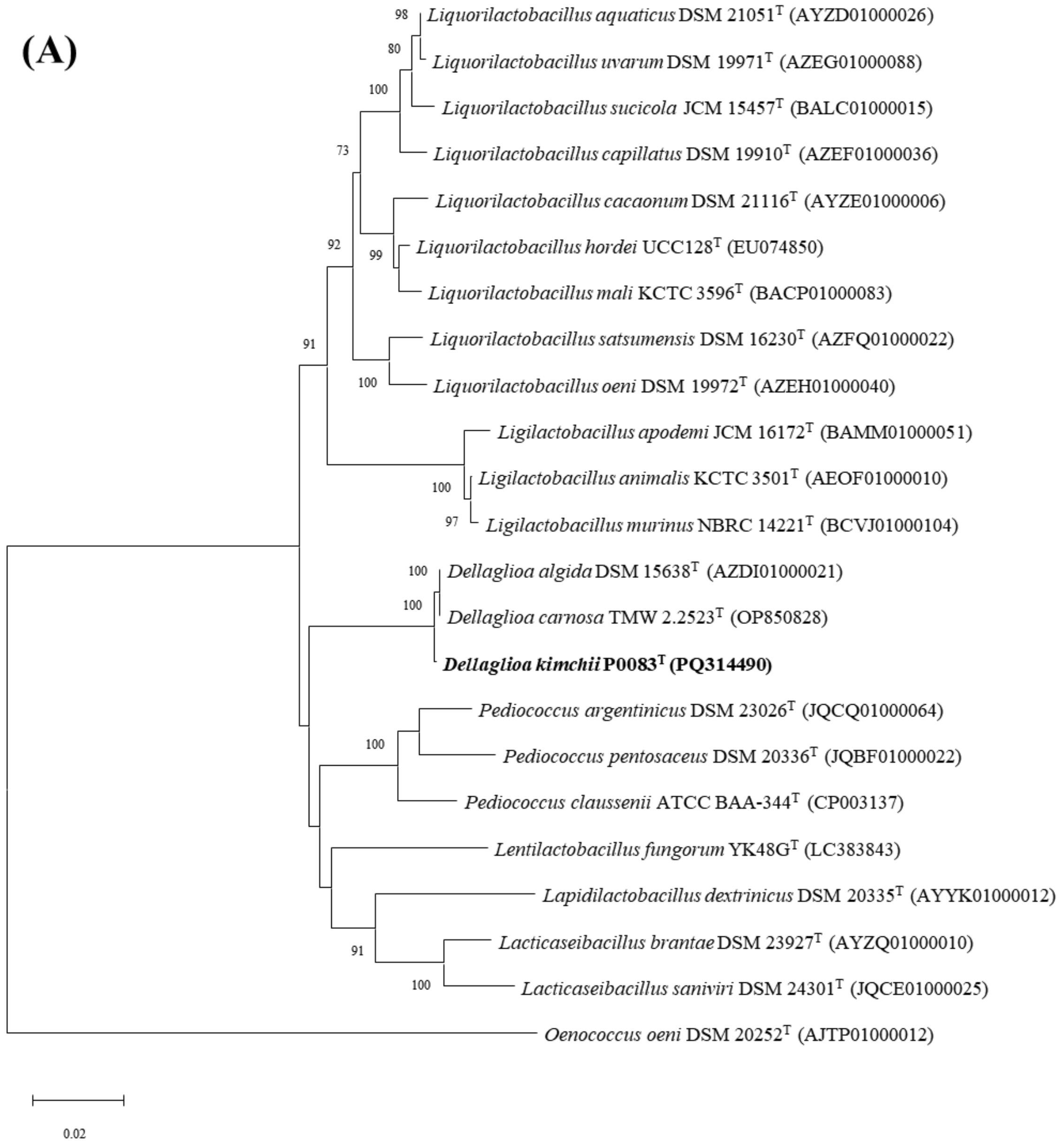

Liquorilactobacillus aquaticus DSM 21051T (AYZD01000026)
Liquorilactobacillus uvarum DSM 19971T (AZEG01000088)
Liquorilactobacillus sucicola JCM 15457T (BALC01000015)
Liquorilactobacillus capillatus DSM 19910T (AZEF01000036)
Liquorilactobacillus cacaonum DSM 21116T (AYZE01000006)
Liquorilactobacillus hordei UCC128T (EU074850)
Liquorilactobacillus mali KCTC 3596T (BACP01000083)
Liquorilactobacillus satsumensis DSM 16230T (AZFQ01000022)
Liquorilactobacillus oeni DSM 19972T (AZEH01000040)
Ligilactobacillus apodemi JCM 16172T (BAMM01000051)
Ligilactobacillus animalis KCTC 3501T (AEOF01000010)
Ligilactobacillus murinus NBRC 14221T (BCVJ01000104)
Dellaglioa algida DSM 15638T (AZDI01000021)
Dellaglioa carnosa TMW 2.2523T (OP850828)
Dellaglioa kimchii P0083T (PQ314490)
Pediococcus argentinicus DSM 23026T (JQCQ01000064)
Pediococcus pentosaceus DSM 20336T (JQBF01000022)
Pediococcus claussenii ATCC BAA-344T (CP003137)
Lentilactobacillus fungorum YK48GT (LC383843)
Lapidilactobacillus dextrinicus DSM 20335T (AYYK01000012)
Lacticaseibacillus brantae DSM 23927T (AYZQ01000010)
Lacticaseibacillus saniviri DSM 24301T (JQCE01000025)
Oenococcus oeni DSM 20252T (AJTP01000012)
98
80
100
73
92
99
91
100
100
97
100
100
100
91
100
0.02

(B)

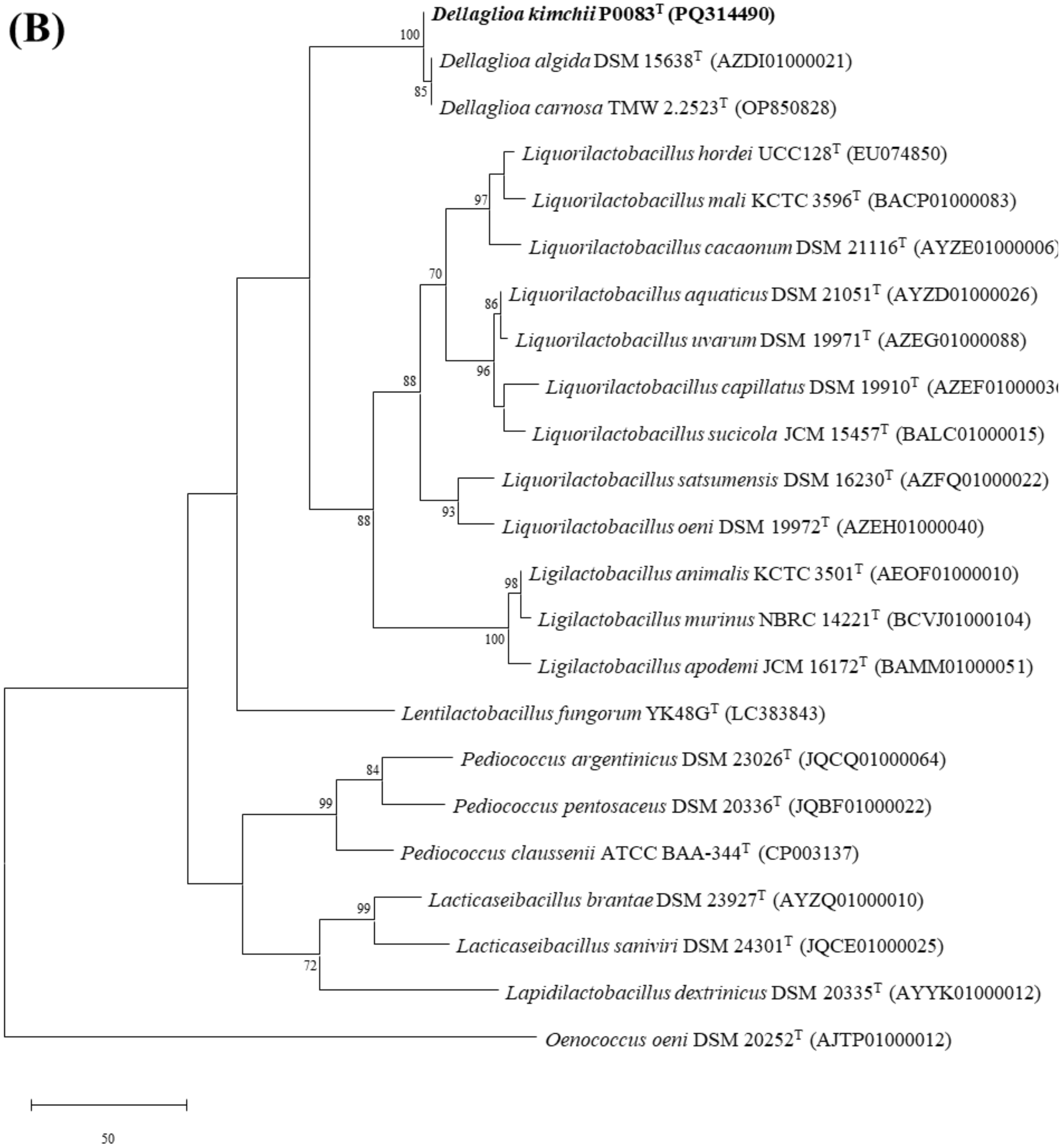

Supplementary Figure 1. Phylogenetic tree of *Dellaglioa kimchii* P0083^T and closely related species using neighbor-joining (A) and maximum parsimony (B) algorithms based on an alignment of 1483 bp nucleotides of 16S rRNA gene sequences. Bootstrap values, calculated from 1,000 replicates, are displayed on the nodes only when 70% or higher. *Oenococcus oeni* DSM 20252^T was used as the outgroup. In neighbor-joining, the scale bar is 0.02 changes per nucleotide position; in maximum parsimony, the scale bar is the units of the number of changes over the whole sequence.

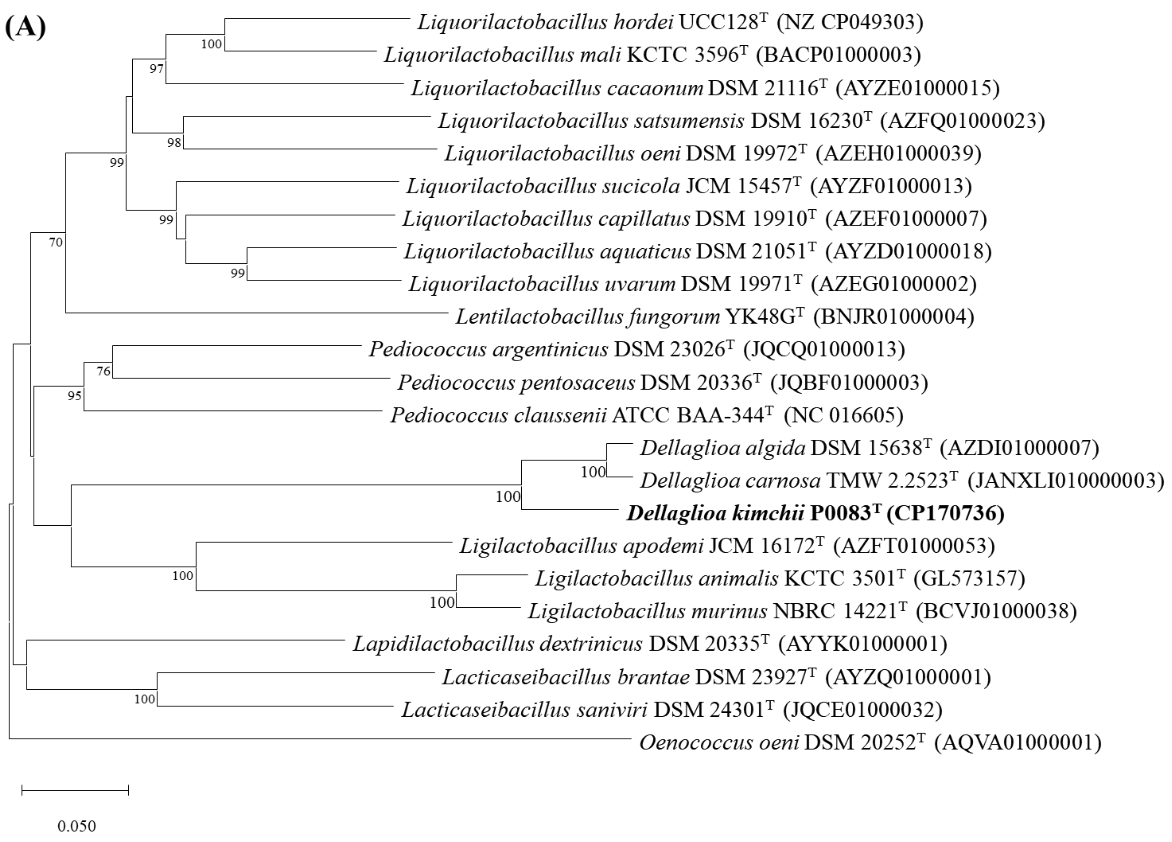
(A)
Liquorilactobacillus hordei UCC128T (NZ CP049303)
Liquorilactobacillus mali KCTC 3596T (BACP01000003)
Liquorilactobacillus cacaonum DSM 21116T (AYZE01000015)
Liquorilactobacillus satsumensis DSM 16230T (AZFQ01000023)
Liquorilactobacillus oeni DSM 19972T (AZEH01000039)
Liquorilactobacillus sucicola JCM 15457T (AYZF01000013)
Liquorilactobacillus capillatus DSM 19910T (AZEF01000007)
Liquorilactobacillus aquaticus DSM 21051T (AYZD01000018)
Liquorilactobacillus uvarum DSM 19971T (AZEG01000002)
Lentilactobacillus fungorum YK48GT (BNJR01000004)
Pediococcus argentinicus DSM 23026T (JQCQ01000013)
Pediococcus pentosaceus DSM 20336T (JQBF01000003)
Pediococcus claussenii ATCC BAA-344T (NC 016605)
Dellaglioa algida DSM 15638T (AZDI01000007)
Dellaglioa carnosa TMW 2.2523T (JANXLI010000003)
Dellaglioa kimchii P0083T (CP170736)
Ligilactobacillus apodemi JCM 16172T (AZFT01000053)
Ligilactobacillus animalis KCTC 3501T (GL573157)
Ligilactobacillus murinus NBRC 14221T (BCVJ01000038)
Lapidilactobacillus dextrinicus DSM 20335T (AYYK01000001)
Lacticaseibacillus brantae DSM 23927T (AYZQ01000001)
Lacticaseibacillus saniviri DSM 24301T (JQCE01000032)
Oenococcus oeni DSM 20252T (AQVA01000001)
100
97
98
99
99
99
70
76
95
100
100
100
100
100
0.050

(B)

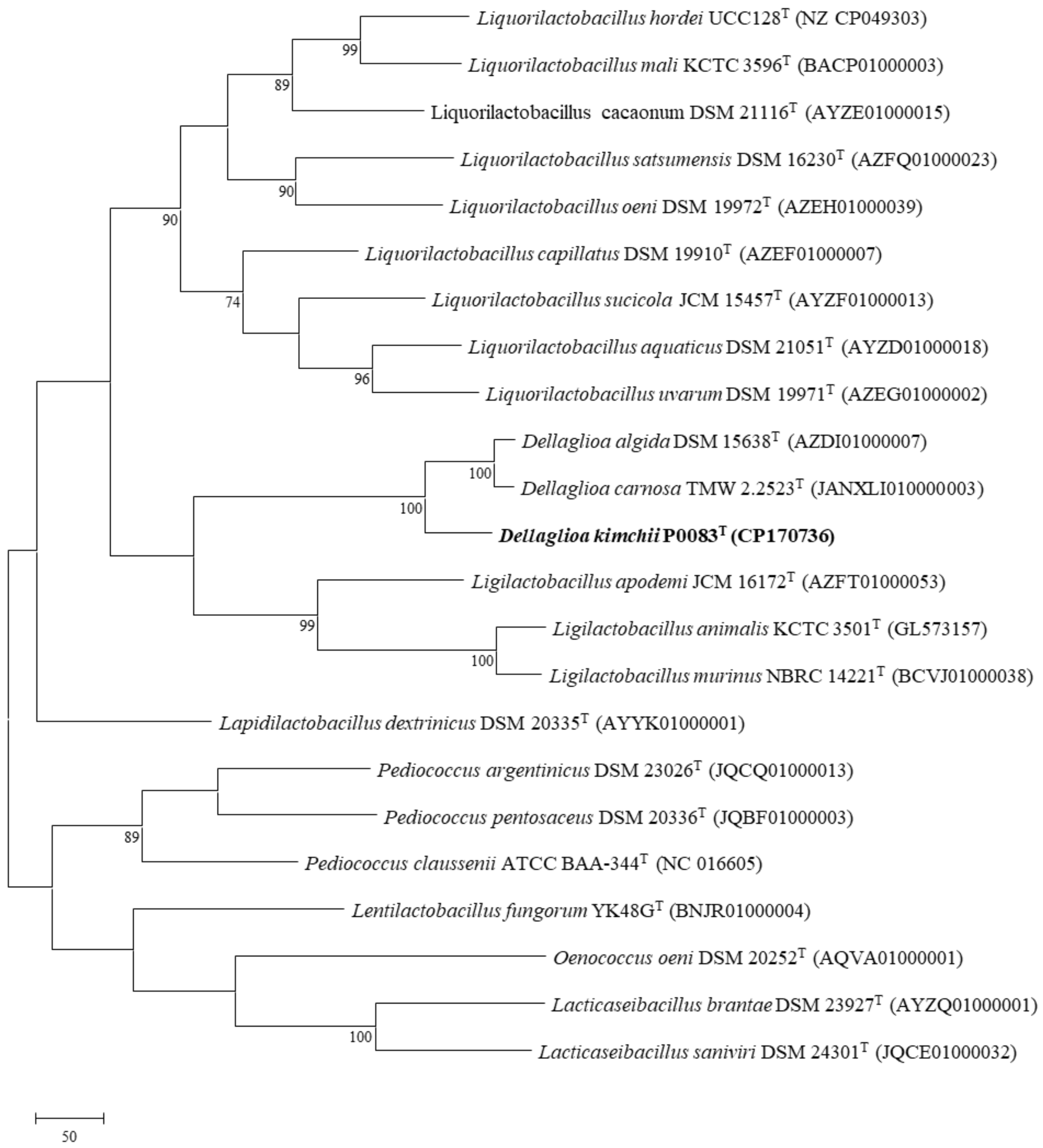

Supplementary Figure 2. Phylogenetic tree of *Dellaglioa kimchii* P0083^{T} and closely related species using neighbor-joining (A) and maximum parsimony (B) algorithms based on *recA* gene sequences. Bootstrap values, calculated from 1,000 replicates, are displayed on the nodes only when 70% or higher. *Oenococcus oeni* DSM 20252^{T} was used as the outgroup. In neighbor-joining, the scale bar is 0.05 changes per nucleotide position; in maximum parsimony, the scale bar is the units of the number of changes over the whole sequence.
